# Demographics and Etiology for Lower Extremity Amputations—Experiences of an University Orthopaedic Center in Germany

**DOI:** 10.3390/medicina59020200

**Published:** 2023-01-19

**Authors:** Annette Eidmann, Yama Kamawal, Martin Luedemann, Peter Raab, Maximilian Rudert, Ioannis Stratos

**Affiliations:** Department of Orthopaedic Surgery, Julius-Maximilians University Wuerzburg, Koenig-Ludwig-Haus, Brettreichstrasse 11, 97074 Wuerzburg, Germany

**Keywords:** lower extremity amputation, major amputation, minor amputation, orthopaedic surgery

## Abstract

*Background and Objectives*: Currently, the worldwide incidence of major amputations in the general population is decreasing whereas the incidence of minor amputations is increasing. The purpose of our study was to analyze whether this trend is reflected among orthopaedic patients treated with lower extremity amputation in our orthopaedic university institution. *Materials and Methods*: We conducted a single-center retrospective study and included patients referred to our orthopaedic department for lower extremity amputation (LEA) between January 2007 and December 2019. Acquired data were the year of amputation, age, sex, level of amputation and cause of amputation. T test and Chi² test were performed to compare age and amputation rates between males and females; significance was defined as *p* < 0.05. Linear regression and multivariate logistic regression models were used to test time trends and to calculate probabilities for LEA. *Results*: A total of 114 amputations of the lower extremity were performed, of which 60.5% were major amputations. The number of major amputations increased over time with a rate of 0.6 amputation/year. Men were significantly more often affected by LEA than women. Age of LEA for men was significantly below the age of LEA for women (men: 54.8 ± 2.8 years, women: 64.9 ± 3.2 years, *p* = 0.021). Main causes leading to LEA were tumors (28.9%) and implant-associated complications (25.4%). Implant-associated complications and age raised the probability for major amputation, whereas malformation, angiopathies and infections were more likely to cause a minor amputation. *Conclusions*: Among patients in our orthopaedic institution, etiology of amputations of the lower extremity is multifactorial and differs from other surgical specialties. The number of major amputations has increased continuously over the past years. Age and sex, as well as diagnosis, influence the type and level of amputation.

## 1. Introduction

Even though lower extremity amputation (LEA) is one of the oldest surgical techniques, dating back to the time of Hippokrates and beyond, it is still part of daily routine for many surgical specialties [1].

Causes leading to LEA differ between countries and geographic regions. In the western world, up to 75% of all LEAs are associated with diabetes mellitus (DM) and peripheral arterial disease (PAD) [2,3,4]. In less developed countries, amputations are more often due to trauma, infection and tumors [5,6,7]. Because of LEA’s high socioeconomic impact as well as the relevant effect on the amputees themselves, efforts have been made to analyze and reduce amputation rates. Population-based data show a trend towards declining rates for major amputations in Europe and worldwide in the last decades, but increasing rates for minor amputations at the same time [2,3,4,8,9,10,11,12,13,14]. Generally, major amputations are defined as amputations proximal to, or through the ankle joint, whereas minor amputations are amputations distal to the ankle joint. Most of the aforementioned studies focus on DM- and PAD-associated lower limb amputations. These diseases represent the largest group and thus have the greatest impact, especially in times of a worldwide increasing prevalence of DM [15]. Other causes for LEA are either excluded in these studies or are not further differentiated. 

In orthopaedic surgery, LEA is also an important part of the surgical practice. In our daily routine, despite the trend to minimal and less-invasive operation techniques, there is an impression that the importance of lower limb amputations, especially of major amputations, had been increasing. As the population is getting older and patients are often multimorbid with a complex surgical history, the options for surgical treatment sometimes are limited to the most radical one, i.e., an amputation. Nevertheless, the main causes leading to LEA in orthopaedic patients tend to be a result of main diagnoses other than DM and PAD. As those diagnoses represent only a small number, regarding the overall population, they are excluded in the existing population-based studies. In the existing literature, no data exist about demographics, etiology and especially time trends of amputations in orthopaedic patients, which are not mainly due to DM and PAD. Therefore, it is unclear if the general trend of decreasing major amputation rates can also be assumed for orthopaedic patients. 

The purpose of this study was to analyze the demographics and etiology for LEA in a collective of orthopaedic patients. Our hypothesis was that, contrary to the general trend, the number of amputations, especially major amputations, had been increasing over time. The results of this single-institution study may be used as a possible indicator for trends in this surgical discipline.

## 2. Materials and Methods

### 2.1. Study Design

The present study is a single-center retrospective study. A total of 106 patients treated between 2007 and 2019 were included.

### 2.2. Study Population

We retrospectively analyzed all digital patient-records of our orthopaedic hospital from amputated patients. To identify these patients, we searched for amputation-specific surgical codes (operating procedure keys (OPS): 5-864.0-9, 5-864.a/x/y, 5-865.0-9 and 5-865.x/y according to the classification of the “German Billing System for Inpatients” (DRG)). All patients who underwent any LEA between January 2007 and December 2019 were included in the study. Year of amputation, age, sex, level of amputation (exarticulation of the hip/transfemoral/exarticulation knee/transtibial/exarticulation ankle/midfoot/metatarsals/toe) and main diagnosis leading to amputation were collected and analyzed. Revisions on the same limb and on the same level of amputation were excluded from the analysis. Any additional amputation on the same patient was counted as separate amputation. As major amputations we defined amputations proximal to, or through the ankle joint, whereas minor amputation was defined as any amputation distal to the ankle joint. Causes for amputations were determined by the main diagnosis directly leading to the amputation according to the DRG system. Secondary diagnoses were not included. Following causes for amputation were classified: tumor, implant-associated complications (e.g., failed total joint replacements and osteosynthesis due to infection or fracture), angiopathies (DM- and PAD-related), malformation, infection (without implant- and DM/PAD-associated infections) and others (e.g., due to trauma). 

### 2.3. Ethics Approval

All data used for analysis were part of the routine medical documentation. Ethical approval was waived by the local Ethics Committee of University of Würzburg in view of the retrospective nature of the study (Protocol number 20210125 02).

### 2.4. Statistics

Results are shown as means and standard error of the mean (S.E.M.). T test and Chi² test were performed to compare age and amputation rates between males and females; significance was defined as *p* < 0.05. To test time trends for major and minor amputations, linear regression was used, followed by likelihood ratio test to test for statistical significance. To calculate probabilities for LEA, multivariate logistic regression models were performed using age, level of amputation and indication as variables. A commercially available and a free statistical software program (Prism 8, GraphPad, CA, USA; jamovi version 1.6.9, the jamovi project (2020)) were used for statistical analysis and data visualization.

## 3. Results

Between 2007 and 2019, 114 amputations of the lower extremity were performed on 106 patients. A total of 60.5% (n = 69) were major and 39.5% (n = 45) were minor amputations. Of these, 61.4% (n = 70) of the patients were male, 38.6% (n = 44) were female. Men were significantly more often affected by LEA than women (*p* = 0.015) and the age at amputation was significantly younger for men than for women (men: 54.8 ± 2.8 years, women: 64.9 ± 3.2 years, *p* = 0.021) (Table 1). 

**Figure 1:**
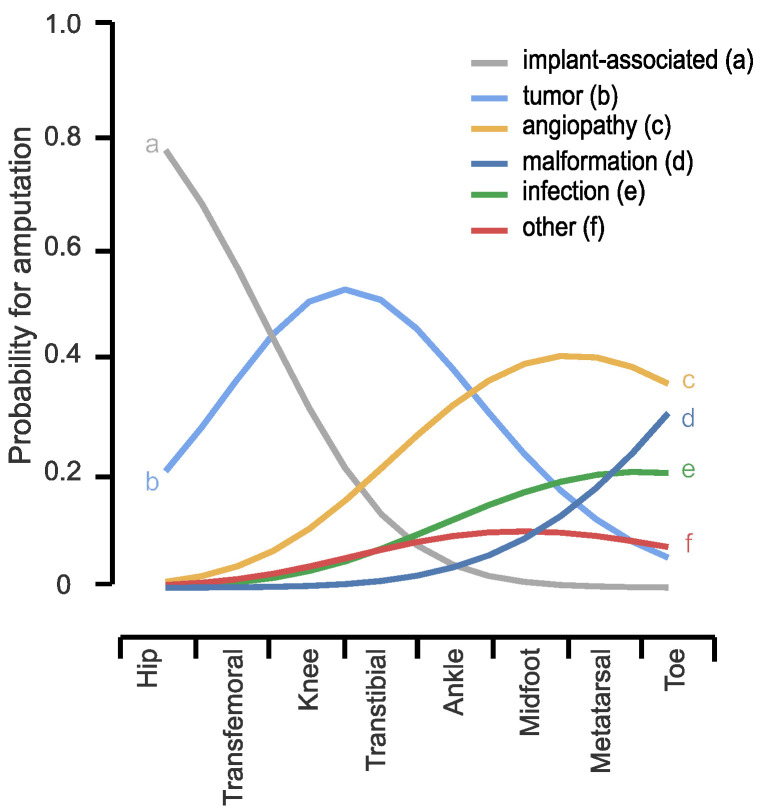
Multivariate logistic regression analysis for the level of amputation: The diagram illustrates the probability for amputation by cause in relation to the level of amputation.

Main causes leading to LEA were tumors (28.9%) and implant-associated complications (25.4%), followed by angiopathies (21.1%), malformations (9.6%), infections (9.6%) and others (5.3%) (Table 2). All implant-associated complications and most tumors led to major amputation. Implant-associated complications increased the probability of a transfemoral amputation and of an exarticulation of the hip. Tumors are associated with a high chance of an amputation around the knee. Malformations, angiopathies and infections are more likely to cause a minor amputation (Figure 1 and Table 3). 

**Figure 2:**
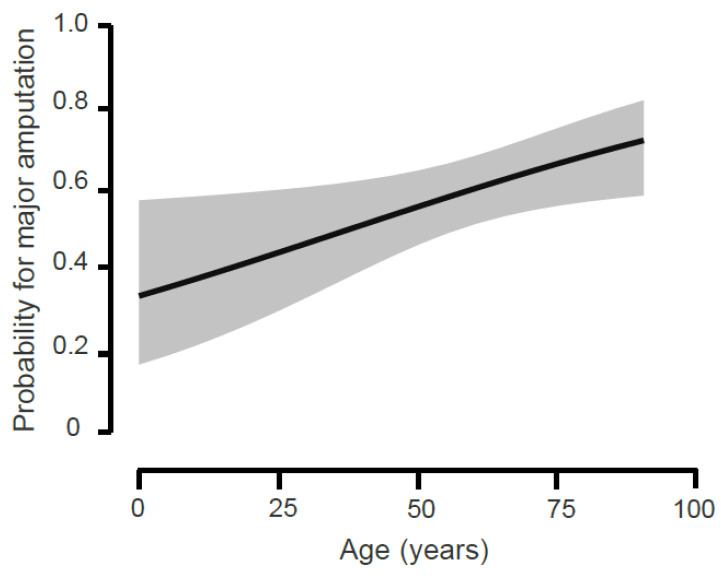
Logistic regression analysis for the probability of major amputation: The diagram shows the probability for a major amputation in relation to age. Conditional estimates plot with 95% confidence interval.

**Figure 3:**
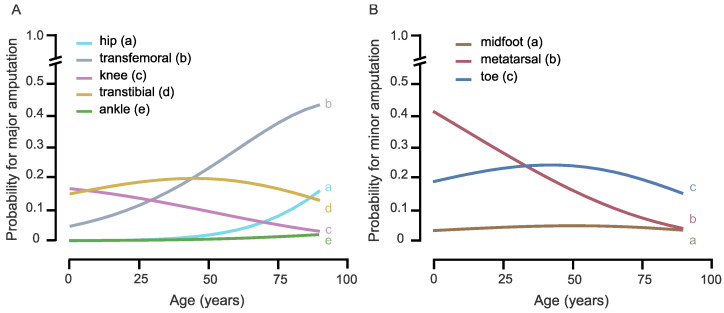
Multivariate logistic regression analysis for the level of amputation: The diagrams illustrate the probability for different levels of amputation in relation to age of amputation. (**A**) probability for major amputation. (**B**) probability for minor amputation.

Increasing age significantly raises the probability of a major amputation, especially of a transfemoral amputation or an exarticulation of the hip (Figure 2 and Figure 3 and Table 3).

**Figure 4:**
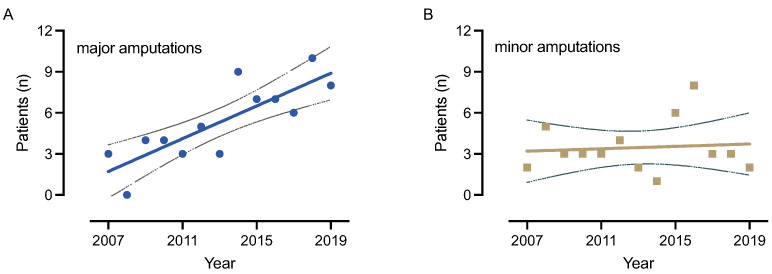
Linear regression analysis for major (**A**) and minor (**B**) amputations over time. During the study period from 20017 to 2019, major amputations are increasing significantly, while minor amputations stay at a constant level. Linear regression equation for major: y = 0.5989x − 1200; R²(major) = 0.675, linear regression equation for minor: y = 0.0440x − 85.02; R²(minor) = 0.008; likelihood ratio test: *p* = 0.0006 for major.

Time trend from 2007 to 2019 shows a significantly increasing number of major amputations (on average 0.6 new major amputations per year) and an almost constant number of minor amputations at the same time (on average 0.04 new minor amputations per year) (Figure 4). 

## 4. Discussion

In this retrospective single-center analysis, we showed that in a cohort of orthopaedic patients, etiology of amputations of the lower extremity is multifactorial and differs from other surgical specialties. Most common diagnoses leading to LEA in our collective of patients are tumors and implant-related complications, in contrast to the worldwide leading position of PAD/DM-related amputations [2,3]. Age and sex, as well as diagnosis, influence the type and level of amputation. Our time trends show an increasing number of major amputations with stable minor amputation rates during the observed period.

Most patients needing LEA in our population were males. Various studies have demonstrated that male sex is a risk factor for amputation, in the diabetic as well as in the non-diabetic population [4,9,11,14,16,17,18,19]. Additionally, males are significantly younger at the time of amputation compared to females as shown in the current study and other population-based studies [4,8,19,20]. This might be explained due to general demographic factors like a higher average age for women as well as lifestyle factors with a higher predisposition for certain comorbidities like DM and PAD [20,21].

Average age for amputation in our study is at 58.7 years. Other studies show 10 to 15 years’ higher mean values [3,11,20]. This difference can be explained due to the fact that these studies analyze patients with overrepresented comorbidities like DM and PAD. These comorbidities are typically found in older patients. Our orthopaedic patients are diagnosed more frequently with musculoskeletal tumors, which can affect children and adolescents, or malformations, which are usually treated by amputation during early infancy. Thus, amputations among orthopaedic patients are not automatically an issue concerning only aged patients.

Nevertheless, increasing age has been shown to be a risk factor for major amputation, especially transfemoral amputation and exarticulation of the hip. This finding in our collective is also in line with other studies [4,11]. Currently, amputation is in most cases not the first line therapy for tumors; most tumor-associated amputations in our collective were due to relapses or complications like failed extremity-preserving techniques. Thus, the probability of amputation rises with age. The same can be assumed for implant-associated amputations. Most of them are salvage procedures after failed total joint replacements, mostly after a multi-annual patient history with multiple previous revision procedures [22]. 

Most studies from Europe and other “western countries” have shown declining rates for major amputations [2,3,4,8,9,10,11,12,13], which is contrary to the increasing major amputations found in our collective. Most amputations in the western world are associated with PAD and DM. Thus, implementing efficient therapies for DM and PAD can lead not only to a decrease of amputation rates in DM/PAD-related amputation rates, but to a decrease in the overall amputation rates. Interventional novelties in the field of angiology, improved recanalization techniques for PAD, better DM disease prevention programs as well as specialized diabetic foot care have led to this success [23,24,25]. Despite this process, there is still need for limb-preserving efforts: the goal of reducing DM-associated amputations by half within five years, which was set in the St. Vincent’s Declaration of 1989, is still not reached [26]. 

According to a recent publication from Kröger et al., a decrease of major amputation rates has been observed in Germany between 2005 and 2014, whereas the overall number of amputations has increased by 3.5%. After exclusion of tumors and other musculoskeletal diseases, the increase of LEA is only 1.1% [8], which implies that musculoskeletal disorders relevantly contribute to LEA. The authors also state particularly that the number of major-amputations have not decreased for patients with tumors and other musculoskeletal disorders. Similar conclusions can be made according to the study from Walter et. al. [4]. In 2019 in Germany, 80% of minor amputations were due to PAD and DM, but only 69.2% of major amputations. This implies that the impact of “other” diagnosis like tumors, musculoskeletal disorders and complications due to implants is higher in the group of major amputations.

In the USA, Kalbaugh et al. have shown a change of indications leading to amputation between 2006 and 2016 [19]. The number of tumors, which are responsible for about 1% of all amputations, has been stable over time, but in the group of “infections”, an increase from 8% to 24% has been detected. 

In our population, the increase of major amputations referred to an increase of implant-related complications. The number of primary total joint replacements in Germany has been at a constant high level in previous years [27], thus leading to an increasing number of revision surgeries, which might rise even more in the future. This is not only due to the high prevalence of protheses, but also to the ageing population [28]. A consequence of increasing revision rates is an increasing number of failed total joint replacements, usually after numerous previous operations and mostly due to chronic infection with loss of function, defects of bone and soft tissue. Thus, it is not surprising that amputations due to periprosthetic complications are increasing, although this remains a salvage procedure. Nevertheless, even small increases should be taken seriously, as major amputation after total joint replacement is a catastrophic procedure for both patient and surgeon. 

Our study has several limitations. We analyzed only a small group of patients which were treated in our institution. Therefore, this sample might not be representative for orthopaedic patients in general due to selection bias. In Germany, LEA are performed by several surgical disciplines. Thus, possible confounders could be regional structural conditions like the specialization of our hospital, the availability of specialized vascular surgeons within reach, as well as changes in procedures or surgical staff. Thus, data represent only a cohort of orthopaedic patients and cannot easily be generalized to the German population. A population-based analysis would be necessary, but using diagnoses and procedure codes from hospital or insurance data makes it difficult if not impossible to differentiate certain groups in the way we did. Nevertheless, musculoskeletal tumors remain rare diseases and consequently, amputations due to tumors are even more sparse. Consequently, in that context, the number of patients reported in this study is not small for a single institution. A good way to enlarge the study population would be a multi-center analysis. 

Because the study population was small, we did not perform age and sex adjustment. Thus, changes in the absolute number of amputations might be due to changes in the affected age groups. Nevertheless, the impact on hospitals and health care systems remains the same.

## 5. Conclusions

Among patients in our orthopaedic institution, etiology of amputations of the lower extremity is multifactorial and differs from other surgical specialties. The number of major amputations has increased continuously over the past years. Age and sex, as well as diagnosis, influence the type and level of amputation. 

## Figures and Tables

**Table 1:** Gender and age distribution of lower extremity amputations. Distribution of gender and age of all LEA, major and minor amputations. Data are given as Means ± S.E.M.; * *p* = level of significance for the comparison female versus male for all LEA (χ² test); ** *p* = level of significance for the comparison female versus male for the factor age (*t* test).

	Female	Male	Total	
Amputations [n]	44	70	114	* *p* = 0.015
Major [n]	28	41	69	
Minor [n]	16	29	45	
Age [years]	64.9 ± 3.2	54.8 ± 2.8	58.7 ± 2.1	** *p* = 0.021

**Table 2:** Causes for lower extremity amputation.

Indication	Major [n]	Minor [n]	Total [n;(%)]
Tumor	29	4	33 (28.9)
Implant-associated complications	29	0	29 (25.4)
Angiopathies	7	17	24 (21.1)
Malformation	0	11	11 (9.6)
Infection	2	9	11 (9.6)
Others	2	4	6 (5.3)

**Table 3:** Model fit measures for logistic regression analysis. Akaike information criterion (AIC), Bayesian information criterion (BIC), McFadden’s pseudo-R2 (R²McF), degrees of freedom (df), probability (*p*). * *p* = level of significance for each comparison (left column) for all LEA (χ² test).

					Overall Model Test		
Model	Deviance	AIC	BIC	R²_McF_	χ²	df	*p*
Age vs. Level of Amputation	392	420	458	0.046	18.9	7	* *p* = 0.009
Cause for amputation vs. Level of amputation	277	297	324	0.261	97.6	5	* *p* < 0.001
Major amputations vs. Age	148	152	157	0.035	5.41	1	* *p* = 0.020

## Data Availability

The datasets generated during the current study are available from the corresponding author on reasonable request.
